# Complete Genome Sequencing of a Community-Associated Methicillin-Resistant Staphylococcus aureus ψUSA300 Strain JICS127, a Uniquely Evolved USA300 Lineage in Japan

**DOI:** 10.1128/mra.00717-22

**Published:** 2022-08-15

**Authors:** Koh Shinohara, Tadashi Baba, Takashi Sasaki, Katsuji Teruya, Yuki Uehara

**Affiliations:** a Department of Clinical Laboratory Medicine, Kyoto University Graduate School of Medicine, Kyoto, Japan; b Department of Microbiology, Juntendo University Faculty of Medicine, Tokyo, Japan; c Graduate School of Nursing, Seisen Jogakuin College, Nagano, Japan; d Animal Research Center, Sapporo Medical University School of Medicine, Sapporo, Japan; e AIDS Clinical Center, the National Center for Global Health and Medicine, Tokyo, Japan; f Department of Infectious Diseases, Fujita Health University School of Medicine, Toyoake, Aichi, Japan; University of Rochester School of Medicine and Dentistry

## Abstract

A ψUSA300 clone of MRSA, a derivative of USA300, is uniquely found in Japan and has 12-bp deletion on *ccrB2* in type IVa staphylococcal cassette chromosome *mec* element. We hereby present the complete genome of ψUSA300 strain JICS127.

## ANNOUNCEMENT

Methicillin-resistant Staphylococcus aureus (MRSA) is a global concern in both community-associated and health care-associated infections (1). The USA300 clone is a predominant MRSA linage in North American countries since early 2000s (2). Although the USA300 clone was rarely isolated in Japan, recent studies have suggested an increased prevalence (3–6). In Japan, ψUSA300 was identified as a USA300-like clone that had a 12-bp deletion on *ccrB2* gene in SCC*mec* (7), and the whole genomic structure has remained unknown. Here, we report the complete genome sequence of a ψUSA300 strain JICS127 isolated from a subcutaneous abscess of an HIV-infected patient in Tokyo. This study was approved by the Human Research Ethics Committee of the National Center for Global Health and Medicine (no. NCGM-G-003353-00).

Strain JICS127 was isolated using mannitol-salt agar (Kyokuto Pharmaceutical Industrial Co., Ltd., Tokyo, Japan) and incubated at 37°C for 48 h. Bacterial culture was grown under the same conditions, and DNA was extracted using a DNA isolation kit ISOPLANT II (Nippon GENE Co., Ltd., Tokyo, Japan). DNA library was constructed using an SMRTbell Express Prep kit v2.0 (PacBio, Menlo Park, CA, USA). Single-molecule real-time (SMRT) sequencing was performed by a PacBio RS II using standard protocols (MagBead Standard Seq v2 loading; 1 × 180-min movie). De novo assembly was performed by HGAP v.3.0. Next, the DNA library was prepared using a Nextera XT DNA sample prep kit (Illumina, San Diego, CA, USA), and sequencing was performed using a MiSeq reagent kit v2 and a paired-end 2 × 250-bp cycle run on the system. To correct sequencing errors, the Illumina reads were mapped to the PacBio contigs and consensus sequences were built using bwa-0.7.5a and samtools-0.1.19 (8, 9). Default parameters were used for all software unless otherwise specified. No genome overlap was identified in a dot plot analysis based on Blast alignment against the reference strain TCH1516 using NCBI Blast Server. The start position of DNA sequence was determined with reference to other complete genome-sequenced S. aureus strains. Gene annotation was performed on the Rapid Annotations using Subsystems Technology (RAST) server (10) and checked and modified manually.

The complete circular genome contains three contigs, consisting of one chromosome of 2,879,025 bp (151-fold coverage) and two plasmids of 40,253 bp (368-fold coverage) and 17,851 bp (265-fold coverage), respectively. The overall G + C content is 32.72%. The chromosome of JICS127 consists of 2,805 genes, of which there are 2,726 coding DNA sequences, 19 rRNA subunits (7 genes for 5S, 6 for 16S, and 6 for 23S), and 60 tRNAs. The two plasmids consist of 60 and 19 coding DNA sequences, respectively. According to both results in mapping using Illumina short reads and assembly using PacBio long read, a 12-bp deletion on the *ccrB2* in the SCC*mec* was identified in the strain JICS127.

This entry into the GenBank database of the strain JICS127 will contribute to investigations into molecular epidemiology and infection control strategies for MRSA.

### Data availability.

The genome sequence of strain JICS127 has been deposited in the GenBank database under accession no. AP025693 (chromosome), AP025694, and AP025695 (plasmids JICSp1 and JICSp2). The raw data from Illumina (DRR227240) and PacBio (DRR374827) have been deposited in the SRA.
